# Sub-Diffraction Photoacoustic Microscopy Enabled by a Novel Phase-Shifted Excitation Strategy: A Numerical Study

**DOI:** 10.3390/s26020498

**Published:** 2026-01-12

**Authors:** George J. Tserevelakis

**Affiliations:** 1 Department of Biology, University of Crete, 700 13 Heraklion, Crete, Greece; tserevel@iesl.forth.gr; 2 Institute of Electronic Structure and Laser, Foundation for Research and Technology Hellas, 700 13 Heraklion, Crete, Greece

**Keywords:** sub-diffraction, photoacoustic, microscopy, low cost, frequency domain

## Abstract

**Highlights:**

**What are the main findings?**

- The proposed phase-shifted Gaussian and donut beam excitation scheme may confine the effective photoacoustic excitation region beyond the conventional optical diffraction limit.
- Numerical simulations show a ~1.42× lateral resolution improvement at an optimal power ratio of 1.16 between the two beams.

**What are the implications of the main findings?**

- It is shown that sub-diffraction photoacoustic microscopy can be achieved using frequency-domain excitation with continuous-wave lasers.
- The method can pave the way for cost-efficient, high-resolution biomedical photoacoustic imaging without nonlinear contrast mechanisms.

**Abstract:**

This numerical simulation study introduces a novel phase-shifted Gaussian and donut beam excitation strategy for frequency-domain photoacoustic microscopy, capable of achieving optical sub-diffraction-limited lateral resolution. We demonstrate that the spatial overlapping of Gaussian and donut beams with π-radian phase-shifted intensity modulation may confine the effective photoacoustic excitation region, substantially reducing the beam-waist-normalized full width at half maximum value from 1.177 to 0.828 units. This effect corresponds to a ~1.42-fold lateral resolution enhancement compared with conventional focused Gaussian beam excitation. Furthermore, the influence of the optical power ratio between the beams was systematically analyzed, revealing an optimal value of 1.16, balancing excitation confinement and side-lobe suppression. Within this framework, the presented simulation results establish a basis for the experimental realization of phase-shifted dual-beam excitation photoacoustic microscopy systems, with a potential impact on high-resolution biomedical imaging of subcellular and microvascular structures using low-cost continuous-wave optical sources such as laser diodes.

## 1. Introduction

Optical-resolution photoacoustic microscopy (OR-PAM) constitutes an emerging and promising imaging modality, providing optical absorption contrast with high sensitivity while using ultrasound detection to generate images with improved penetration of scattering tissues compared to pure optical microscopy approaches [1]. In OR-PAM, lateral resolution is governed by the tight focusing of an optical beam, leading to the generation of photoacoustic waves composed of frequencies typically in the tens of MHz range. In this context, the technique enables label-free visualization of endogenous chromophores such as hemoglobin, melanin, lipids, and nucleic acids, thus supporting anatomical, functional, flow-dynamic, and molecular imaging in biomedical research and preclinical studies [2]. Despite its advantages, the maximum attainable lateral resolution of OR-PAM is fundamentally restricted by the diffraction-limited focus of the commonly employed Gaussian beam, constraining resolvable feature sizes to approximately 200–250 nm, even when high-numerical-aperture objective lenses are used [1,2]. As a result, this performance barrier renders the technique insufficient for imaging several subcellular components and fine microvascular structures, thereby reducing its applicability in resolution-critical biomedical observations.

To face this challenge, several studies have attempted to surpass this optical diffraction limit in OR-PAM, mostly by exploiting nonlinear effects related to the generation of photoacoustic signals, including optical absorption saturation and thermally induced nonlinearities, to compress the effective point spread function and achieve nanoscale imaging performance. Specifically, the label-free photoacoustic nanoscopy work by Danielli et al. demonstrated sub-diffraction performance through nonlinear dependence of photoacoustic amplitude on excitation conditions [3], while subsequent studies and reviews further clarified how saturation-based mechanisms and related nonlinear effects can be engineered for resolution enhancement and optical sectioning [4,5,6,7]. Nevertheless, these methods typically require extremely high optical fluences to generate nonlinear photoacoustic responses, raising concerns regarding photodamage and phototoxicity issues in sensitive biological specimens. Furthermore, nonlinear methods often rely on the realization of multiple measurements, e.g., by varying pulse energy, significantly increasing the total image acquisition time, as well as the probability of motion artifacts. Since the experimental realization of nonlinear super-resolution photoacoustic microscopy requires highly stable pulsed laser sources together with complex synchronization and detection electronic equipment, such imaging systems tend to be expensive and bulky, thus limiting their applicability in cost-efficient implementations, where simplicity, robustness, and affordability are key design considerations.

During the previous years, continuous-wave (CW) laser diode sources have been investigated as practical photoacoustic excitation sources enabling the emission of short pulses by integrating custom-made drivers or frequency-domain operation through continuous intensity modulation with reduced system footprint and cost [8]. In this direction, diode-based OR-PAM platforms have been positioned as promising routes towards affordable and miniaturizable systems, including demonstrations that emphasize performance, tunability, and translation potential [9,10]. Frequency-domain photoacoustic microscopy (FD-PAM) is a specific class of approaches that typically excite single-frequency photoacoustic waves through sinusoidally intensity-modulated optical radiation while detecting the amplitude and phase of the generated signals [11]. Recent implementations in the literature have demonstrated that FD-PAM can provide near optical diffraction-limited lateral resolution, which can also be combined with traditional optical microscopy methods (e.g., fluorescence detection) to provide complementary contrast in various applications [12,13]. However, despite the promising capabilities of FD-PAM, there has been limited progress in enhancing its resolution performance, particularly in the context of cost-efficient imaging for resolving fine biological structures.

In this study, we introduce and numerically evaluate a novel phase-shifted dual-beam excitation strategy for FD-PAM, exploiting the spatial overlap of a focused Gaussian and a donut-shaped beam. Our simulations show that by driving the intensity of the two beams with sinusoidal modulation at the same frequency but with a π-radian phase offset, the effective photoacoustic excitation region is substantially confined beyond the Gaussian focus alone, enabling sub-diffraction lateral resolution. The proposed concept is fully compatible with practical FD-PAM instrumentation, and it is intended as a low-cost pathway towards resolution-enhanced platforms, significantly expanding the applicability of the method to demanding biomedical imaging applications where diffraction-limited resolution constitutes a key bottleneck.

## 2. Theory and Methods

### 2.1. Photoacoustic Equation Model

The generation of a photoacoustic signal under sinusoidally intensity-modulated optical excitation has been modeled using the time-independent inhomogeneous Helmholtz equation. Assuming thermal and stress confinement conditions, the three-dimensional frequency-domain photoacoustic pressure $p\left(r,\omega \right)$ at position *r* and angular frequency *ω* in an acoustically homogeneous infinite medium satisfies [14,15]

(1) $$\left(\nabla^{2}+k^{2}\right)p\left(r,\omega \right)=\frac{-i\omega \beta }{C_{p}}H(r,\omega )$$

where k is the acoustic wavenumber given by the equation $k=\omega /u_{a}$ ($u_{a}$ stands for the acoustic speed of sound), β is the volumetric thermal expansion coefficient, $C_{p}$ is the specific heat capacity at constant pressure, and H(r,ω) represents the source term driving photoacoustic pressure generation and corresponds to the absorbed optical energy density of the Fourier component of heating at frequency ω. Therefore, under these assumptions, the spatial distribution of the photoacoustic signal amplitude is directly proportional to the absorbed optical energy density at the modulation frequency [14,16].

### 2.2. Optical Excitation Scheme

Two non-interfering optical beams of identical wavelength have been considered for the excitation of photoacoustic signals: a fundamental TEM_00_ Gaussian beam and a helical Laguerre–Gaussian (LG_01_) donut-shaped beam. The beams are assumed to be coaxially aligned and confocally focused by the same objective lens, resulting in identical beam waists ($w_{0}$) at the focal plane (z = 0). Both beams are sinusoidally intensity-modulated at the same frequency *ω*, with the donut beam phase-shifted by *π* radians relative to the Gaussian beam. In this case, the time-dependent intensity distributions are expressed as [12]

(2) $$I_{G}(r,t)=I_{G}(r)[1+mcos\;\omega t]$$

(3) $$I_{D}(r,t)=I_{D}(r)\;[1+mcos\;(\omega t+\pi )]=I_{D}(r)[1-mcos\;\omega t]$$

where $I_{G}(r)$ and $I_{D}(r)$ denote the average spatial intensity profiles of the Gaussian and donut beams, respectively, and m stands for the modulation depth, defined as

(4) $$m=\frac{I_{max}-I_{min}}{I_{max}+I_{min}}$$

with $I_{max}$ and $I_{min}$ representing the maximum and minimum optical intensity values of the modulated beam.

### 2.3. Intensity Distributions

The average Gaussian beam intensity at the focal plane (z = 0) has been modeled as [17]

(5) $$I_{G}\left(r\right)=\frac{2P_{G}}{\pi w_{0}^{2}}e^{-\frac{2r^{2}}{w_{0}^{2}}}\;$$

where $P_{G}$ is the optical power of the beam, and *r* stands for the radial coordinate. The field amplitude of Laguerre–Gaussian beams at z = 0 in cylindrical coordinates is expressed as

(6) $$E_{p,l}\left(r,\theta \right)\propto {\left(\frac{\sqrt{2}r}{w_{0}}\right)}^{\left|l\right|}L_{p}^{\left|l\right|}\left(\frac{2r^{2}}{w_{0}^{2}}\right)e^{-\frac{r^{2}}{w_{0}^{2}}}\;e^{il\theta }$$

where $w_{0}$ corresponds to the beam waist radius, $L_{p}^{\left|l\right|}$ is the associated Laguerre polynomial, and p and l represent the radial and azimuthal indices, respectively [18]. For the simplest and most commonly utilized donut beam in super-resolution microscopy (p=0 and l=1) [19], $L_{0}^{1}=1$, and in this case, the field amplitude can be written as

(7) $$E_{0,1}\left(r,\theta \right)\propto \frac{\sqrt{2}r}{w_{0}}e^{-\frac{r^{2}}{w_{0}^{2}}}\;e^{i\theta }$$

yielding an intensity distribution $I_{D}(r)$ which has the following form:

(8) $$I_{D}\left(r\right)\propto \frac{2r^{2}}{w_{0}^{2}}e^{-\frac{2r^{2}}{w_{0}^{2}}}\;$$

We then introduce a normalization factor *A* and demand that the intensity of the donut beam integrates to the respective optical power $P_{D}$ through the following equation:

(9) $$P_{D}=\;A\int_{0}^{2\pi }\int_{0}^{\infty }\frac{2r^{2}}{w_{0}^{2}}e^{-\frac{2r^{2}}{w_{0}^{2}}}rdrd\theta$$

The analytical calculation of the integral determines the normalization factor as

(10) $$A=\frac{2P_{D}}{\pi w_{0}^{2}}$$

Therefore, the average intensity distribution of the donut beam can be finally written as

(11) $$I_{D}\left(r\right)=\frac{4P_{D}}{\pi w_{0}^{4}}r^{2}e^{-\frac{2r^{2}}{w_{0}^{2}}}\;$$

### 2.4. Effective Photoacoustic Excitation Region

The total absorbed optical power density at the focal plane H(r,t) is obtained in the time domain by taking the product of the medium’s optical absorption coefficient $\mu_{a}$ and the total intensity $I_{G}\left(r,t\right)+I_{D}\left(r,t\right)$ on the absorber [16] through the equation

(12) $$H(r,t)=\mu_{a}[I_{G}(r)+I_{D}(r)]\;+\;\;\mu_{a}m\;[I_{G}(r)-I_{D}(r)]cos\;\omega t\;$$

The absorbed optical energy density H(r,ω) driving the photoacoustic pressure $p\left(r,\omega \right)$ in the Helmholtz equation (Equation (1)) will then be proportional to the Fourier amplitude of H(r,t) at angular frequency *ω*, and thus,

(13) $$\left|p\left(r,\omega \right)\right|\;\propto \;\mu_{a}m\left|I_{G}(r)-I_{D}(r)\right|$$

Therefore, under the conditions described above, the spatial distribution of the photoacoustic signal strength at *ω* will scale with the absolute difference between the average intensities of the two focused beams at each point. ChatGPT 5.2 was used as an auxiliary tool during the development of MATLAB R2018b scripts implementing the equations of the theoretical model for phase-shifted photoacoustic excitation. Specifically, it assisted with code drafting, syntax checking, and optimization suggestions based on author-provided instructions.

## 3. Results

### 3.1. Simulation of Lateral Resolution Enhancement

**Figure 1:**
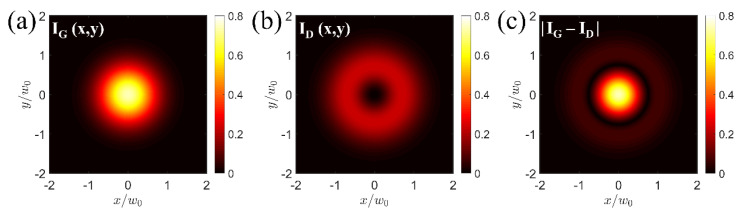
(**a**) Average Gaussian beam intensity distribution for $P_{G}=1.16$. (**b**) Average donut beam intensity distribution for the same $w_{0}$ as in (**a**) and $P_{D}=1$. (**c**) Absolute intensity difference between the two beams representing the resulting point spread function.

Figure 1a,b present the two-dimensional average intensity distribution of a modulated Gaussian (Equation (5)) and a donut-shaped beam (Equation (11)), respectively, using beam-waist-normalized Cartesian coordinates. The beams exhibit the same waist value ($w_{0}=1$ for simplicity), as they are assumed to have identical optical wavelengths and are focused by the same objective lens. The optical power ratio $P_{G}/P_{D}$ has been set to 1.16:1, such that the distribution of the beams’ absolute intensity difference, which reflects the magnitude of the photoacoustic signal according to Equation (13), displays a clearly defined central region, with ring artifacts suppressed by convention to just below 10% of the peak intensity (Figure 1c).

**Figure 2:**
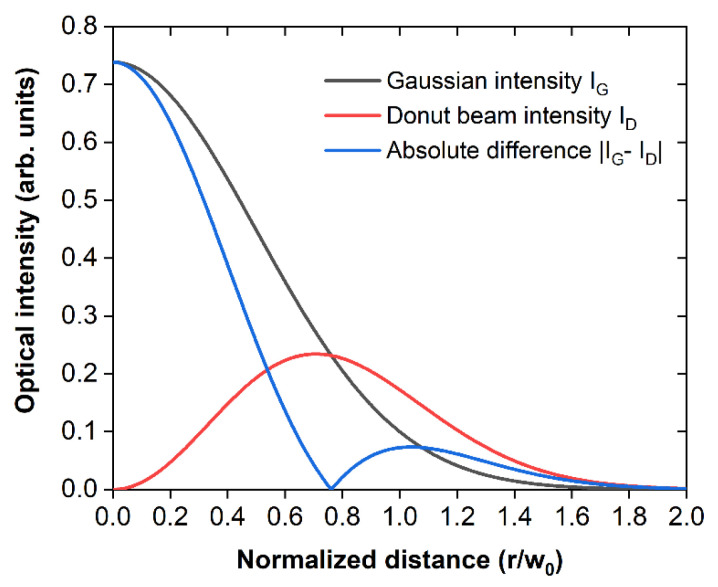
Radial intensity profiles of the simulated Gaussian (black) and donut (red) beams and their absolute difference (blue).

The corresponding radial profiles of the intensity maps presented in Figure 1 demonstrate a substantial reduction in the full width at half maximum (FWHM) value of the absolute intensity difference curve compared to the Gaussian beam’s profile (Figure 2). Specifically, the Gaussian beam’s FWHM is determined at 1.177 normalized units, whereas the respective value for the effective photoacoustic signal generation region (Figure 2; blue curve), defined by the absolute intensity difference between the Gaussian (Figure 2; black curve) and the donut-shaped beam (Figure 2; red curve), is estimated at 0.828 units. Neglecting the minor blurring contribution of the weak-intensity side-lobe, this reduction in the effective point spread function could improve the lateral resolution of a frequency-domain photoacoustic microscope by a factor of ~1.42.

### 3.2. Temporal Evolution of the Excitation Process

**Figure 3:**
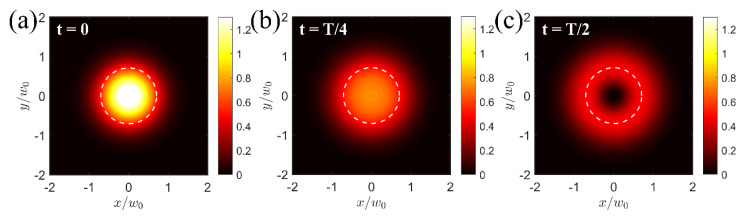
Snapshots of the total intensity distribution $I_{G}\left(r,t\right)+I_{D}\left(r,t\right)$ when t = 0 (**a**), t = T/4 (**b**), and t = T/2 (**c**). The dashed circle indicates the peak intensity of the donut beam.

Figure 3 provides a visualization of the total intensity distribution at the focal plane during half of the excitation cycle through the two simulated intensity-modulated beams, assuming perfect modulation conditions (m = 1). At t = 0 (Figure 3a), the maximum intensity of the Gaussian beam is observed, whereas the donut beam’s intensity is zero. When t = T/4 (Figure 3b), both beams simultaneously illuminate the optical absorber at half of their maximum intensity, providing a smoother optical intensity map. Finally, when t = T/2 (Figure 3c), the Gaussian beam’s intensity is exactly zero, while the donut beam presents its maximum intensity. The excitation process continues with intensity profiles similar to those in Figure 3b for t = 3T/4 and Figure 3a for t = T (Supplementary Video S1). These excitation snapshots clearly demonstrate that the effective intensity modulation responsible for photoacoustic signal production originates predominantly from the center of the “hole” region of the donut beam, indicated by the dashed circle at $r=w_{0}/\sqrt{2}$, corresponding to the peak intensity of the donut profile. On the contrary, the effective modulation depth is substantially suppressed in the peripheral regions of the two overlapping beams, outside of the dashed circle, leading to minimal photoacoustic signal emissions.

### 3.3. Influence of the Optical Power Ratio

**Figure 4:**
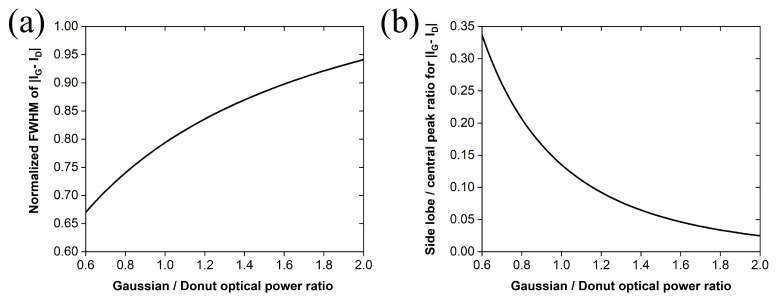
(**a**) Normalized FWHM of the effective excitation region as a function of the optical power ratio between the two beams. (**b**) Normalized side-lobe intensity relative to the central peak, as a function of the applied optical power ratio.

In the next step, we investigated the performance of the phase-shifted photoacoustic excitation scheme across an indicative range of applied average optical power ratios $P_{G}/P_{D}$ between 0.6 and 2. The normalized FWHM of the effective photoacoustic excitation region, defined by the absolute intensity difference between the beams, increases nonlinearly with the power ratio (Figure 4a), thus reducing the achievable lateral resolution as the Gaussian beam dominates. At very high ratios, the curve asymptotically approaches 1.177, consistent with the normalized FWHM of a Gaussian intensity profile. Although lower power ratios favor improved lateral resolution, this benefit is counterbalanced by stronger ring artifacts, whose relative intensity increases with decreasing ratio. This effect is illustrated in the plot of Figure 4b depicting the maximum side-lobe intensity, normalized to the central peak of the absolute intensity difference curve, as a function of the optical power ratio. The two graphs reveal a clear trade-off: ratios below unity increase ring artifacts despite producing more confined photoacoustic excitation regions, whereas higher ratios (~1.6 or above) suppress artifacts but broaden the effective excitation region, with both cases ultimately not providing an optimal lateral resolution. Therefore, the selected power ratio of 1.16 shown in the previous figures can be considered a favorable solution, as it provides a balanced compromise between minimizing ring artifacts and preserving sufficient confinement of the central excitation region to maintain a considerable improvement in the lateral resolution of a photoacoustic microscope.

### 3.4. Phase-Shifted Photoacoustic Imaging Simulation

**Figure 5:**
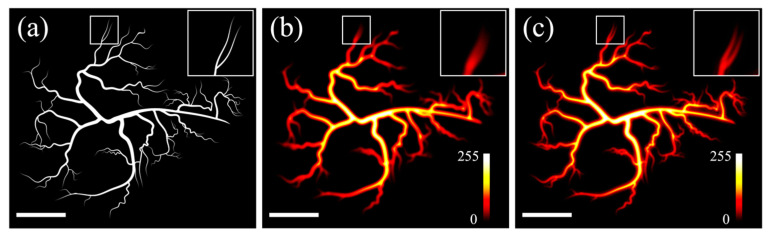
(**a**) The ground-truth microvasculature image. (**b**) A simulated photoacoustic image obtained by convolving the ground-truth image shown in (**a**) with the diffraction-limited PSF of a Gaussian beam (FWHM ≈ 142 nm). The inset shows strong blurring and loss of resolvable branching. (**c**) A simulated image obtained using the proposed phase-shifted excitation scheme with an optical power ratio of 1.16 (central lobe’s FWHM ≈ 100 nm). Enhanced vessel definition and clearly distinguishable branching can be clearly observed. All scalebars are equal to 2 μm.

To further evaluate the practical imaging performance of the proposed phase-shifted excitation strategy in realistic scenarios, we performed numerical scanning simulations using a synthetic microvasculature-like phantom. This analysis complements the previous lateral resolution investigation (see Section 3.1) by demonstrating the impact of the modified excitation profile on image formation and detail recovery. Figure 5a shows the synthetic microvasculature image used as the ground-truth object. The phantom contains vessels of varying widths and branching angles, highly representative of typical realistic microvascular structures, although at much lower dimensions. The inset on the top right highlights a region of interest measuring 1.12 μm^2^ × 1.12 μm^2^, focusing on a bifurcating vessel junction where high-resolution requirements are particularly evident. To simulate conventional Gaussian excitation, the ground-truth image was convolved with the point spread function (PSF), corresponding to a tightly focused Gaussian beam at an indicative wavelength of λ = 532 nm and a lens numerical aperture (NA) equal to 1.4, which is typical for oil-immersion microscopy objectives. Under these conditions, the diffraction-limited FWHM of the excitation spot is approximately equal to 142 nm [17]. The resulting image is shown in Figure 5b. As expected, the microvascular features appear substantially blurred, with pronounced loss of high spatial frequency information. In particular, the corresponding inset demonstrates that closely spaced vessel branches cannot be resolved and merge into a single broadened structure. Figure 5c presents the corresponding simulated image obtained using the proposed phase-shifted excitation scheme, employing the same optical wavelength and NA and an optimal power ratio of 1.16. In this case, the central lobe of the resulting PSF (Figure 1c) presents an apparent FWHM reduction of a factor of approximately 1.42 relative to the conventional Gaussian excitation, yielding a value of around 100 nm. This apparent improvement in spatial confinement of the photoacoustic signal generation leads to a significant enhancement of microvascular detail: vessel boundaries appear sharper, and the branching structure highlighted in the inset becomes clearly distinguishable.

**Figure 6:**
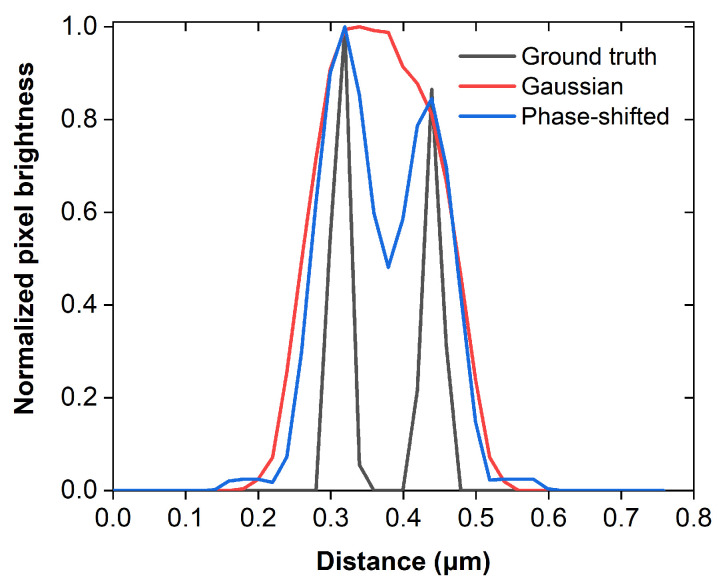
Normalized pixel brightness profiles across the microvascular branch shown in the insets of Figure 5a–c.

The resolution improvement is quantitatively illustrated in Figure 6, which shows normalized brightness profiles extracted across the central part of the vessel branch indicated in the insets of Figure 5a–c. The profile corresponding to Gaussian excitation (red line) exhibits a single broad peak, indicating insufficient resolving capability. In contrast, the phase-shifted excitation profile (blue line) reveals two distinct signal maxima, fully consistent with the separation of adjacent vessel branches in the ground-truth image (black line). This result numerically confirms that the proposed excitation strategy can improve the photoacoustic microscope’s resolving power, enabling discrimination of sub-diffraction vascular features that are otherwise blurred under conventional Gaussian illumination.

## 4. Discussion

This numerical simulation study has demonstrated the potential of a phase-shifted dual-beam excitation scheme for obtaining sub-diffraction lateral resolution in FD-PAM by confining the effective photoacoustic excitation region. Specifically, we have shown that the spatial overlap of a Gaussian and a donut-shaped beam driven by the π-radian phase difference in their intensity modulation may provide a ~1.42-fold resolution enhancement relative to standard Gaussian excitation. This improvement is achieved within the linear photoacoustic excitation regime using standard modulation of laser diodes, without requiring high optical fluences, expensive pulsed laser sources, or complex equipment and synchronization procedures. Therefore, the method could enable high-resolution observations while significantly reducing the complexity, cost, and footprint of photoacoustic microscopy platforms.

It has to be mentioned that the predicted resolution enhancement is more moderate compared to nonlinear methods exploiting absorption saturation or thermally driven nonlinearities, which can lead to increased phototoxicity risk and longer image acquisition times. The proposed excitation strategy could be seen as a trade-off, offering a meaningful improvement that is more compatible with practical, cost-efficient implementations, expanding both the applicability and accessibility of sub-diffraction photoacoustic microscopy methods. Signal-to-noise ratio (SNR) considerations are also particularly relevant in FD-PAM, since single-frequency excitation is known to be less efficient in comparison with nanosecond pulses [12]. In the presented method, part of the optical power is redistributed into the donut beam, and partial cancellation of modulation takes place in peripheral regions, which is expected to reduce the detected absolute photoacoustic signal amplitude. In particular, let *γ* = *P_G_*/*P_D_* denote the optical power ratio between the two beams used in the phase-shifted excitation scheme and define the normalized coordinate x = 2*r*^2^/*w_0_*^2^. We assume that photoacoustic signal generation is confined to the 1/*e* effective excitation region for both the conventional focused Gaussian beam and the phase-shifted excitation approach. For a conventional focused Gaussian beam, the 1/*e* radius can be obtained directly from Equation (5) as

(14) $$r_{1/e,G}=\frac{w_{0}}{\sqrt{2}}$$

The total absorbed energy within the 1/*e* radius of the Gaussian beam is proportional to

(15) $$S_{1/e,G}=\int_{0}^{r_{1/e,G}}I_{G}(r)2\pi rdr=P_{G}(1-e^{-1})$$

For the phase-shifted excitation scheme, the corresponding intensity difference within the 1/*e* radius $\left(r<r_{1/e,\Delta I}\right)$ is obtained by subtracting Equation (11) from Equation (5), yielding

(16) $$\Delta I\left(r\right)=\frac{2P_{D}}{\pi w_{0}^{2}}(\gamma -x)e^{-x}$$

Analogously to the Gaussian excitation case, the total absorbed energy within the 1/*e* radius will be proportional to the integral

(17) $$S_{1/e,\Delta I}=P_{D}\int_{0}^{x_{\Delta I}}(\gamma -x)e^{-x}dx$$

where $x_{\Delta I}$ corresponds to the value of *x* at the radius for which the intensity drops to 1/*e* of its maximum value. Analytical evaluation of the integral in Equation (17) yields

(18) $$S_{1/e,\Delta I}=P_{D}[\left(x_{\Delta I}+1-\gamma \right)e^{-x_{\Delta I}}-\left(1-\gamma \right)]$$

Assuming that the experimental noise level remains unchanged for both excitation schemes, the SNR ratio can be accurately approximated by the ratio of the absorbed energies given in Equations (18) and (15), namely,

(19) $$\frac{S_{1/e,\Delta I}}{S_{1/e,G}}=\frac{\left(x_{\Delta I}+1-\gamma \right)e^{-x_{\Delta I}}-\left(1-\gamma \right)}{\gamma (1-e^{-1})}$$

For the demonstrated power ratio *γ* = 1.16, and $x_{\Delta I}=2{\left(0.704r_{\frac{1}{e},G}\right)}^{2}/w_{0}^{2}\approx 0.496$, where the factor 0.704 corresponds to the previously estimated ∼1.42× lateral resolution improvement, Equation (19) yields a value of approximately 0.50. This corresponds to a moderate SNR penalty of about 6 dB, which can be mitigated through narrowband lock-in detection, longer integration times, or signal averaging. Since the noise spectral density at the modulation frequency is largely independent of the excitation profile, the proposed method remains fully compatible with practical FD-PAM implementations while providing a substantial improvement in lateral resolution. Nevertheless, future experimental investigations will be required to quantitatively assess SNR trade-offs under realistic noise conditions and to identify optimal operating regimes.

An additional factor that could influence the practical performance of the proposed method is the validity of the thermal confinement condition, which was assumed in the context of this study. When thermal confinement conditions are met, the thermal diffusion across the excitation region can be considered negligible during a modulation cycle, ensuring that all absorbed optical energy contributes locally to photoacoustic pressure generation. In FD-PAM, this condition is generally satisfied when sufficiently high modulation frequencies are employed (tens of MHz) for typical beam focusing conditions. A partial violation of the thermal confinement condition could occur at lower modulation frequencies or in media with high thermal diffusivity, leading to spatial blurring of the effective signal excitation area. To provide a basic quantitative analysis of potential thermal diffusion effects, we introduce the characteristic thermal diffusion (penetration) length *L* obtained from solutions of the heat diffusion equation under harmonic excitation, which is given by the following equation [20]:

(20) $$L=\sqrt{\alpha /(\pi f)}$$

Here, *α* denotes the thermal diffusivity of the medium, and *f* corresponds to the modulation frequency of the optical excitation. For soft biological tissues, the thermal diffusivity has a typical value of *α* = 1.3 × 10^−7^ m^2^/s [16]. Solving Equation (20) for *f* yields

(21) $$f(MHz)\approx \frac{4.14\times 10^{4}}{{L(nm)}^{2}}$$

Substituting *L* with the characteristic FWHM value of the effective photoacoustic excitation region for λ = 532 nm, numerical aperture NA = 1.4, and a lateral improvement factor of 1.42 relative to a focused Gaussian beam, we obtain *L* ≈ 100 nm (see Section 3.4). Equation (21) then yields a corresponding modulation frequency of approximately 4.14 MHz. Consequently, to satisfy thermal confinement conditions, the modulation frequency should be significantly higher than this value. Since experimental FD-PAM systems have been successfully demonstrated to operate at modulation frequencies in the range of 10–50 MHz [11], we anticipate that PSF broadening due to thermal diffusion effects will be negligible in practice, particularly for modulation frequencies of 20 MHz and above.

Furthermore, it has to be mentioned that the photoacoustic signal amplitude scales linearly with the optical modulation depth m, as indicated by Equation (13). Throughout this study, ideal modulation conditions (m = 1) have been assumed for clarity. In practice, however, real laser diodes and acousto-optic modulator systems rarely achieve unity modulation depth at high modulation frequencies. Recent FD-PAM implementations have reported modulation depths of approximately m ≈ 0.95 at modulation frequencies of around 10 MHz [13]. At higher frequencies, particularly above 30 MHz, a further reduction in modulation depth is expected due to response limitations of the modulation hardware. Although reduced modulation depth leads to a lower signal amplitude and SNR, this effect is partially compensated by the increased efficiency of photoacoustic signal generation at higher modulation frequencies, as indicated by the frequency-dependent source term of the inhomogeneous Helmholtz equation (Equation (1)).

**Figure 7:**
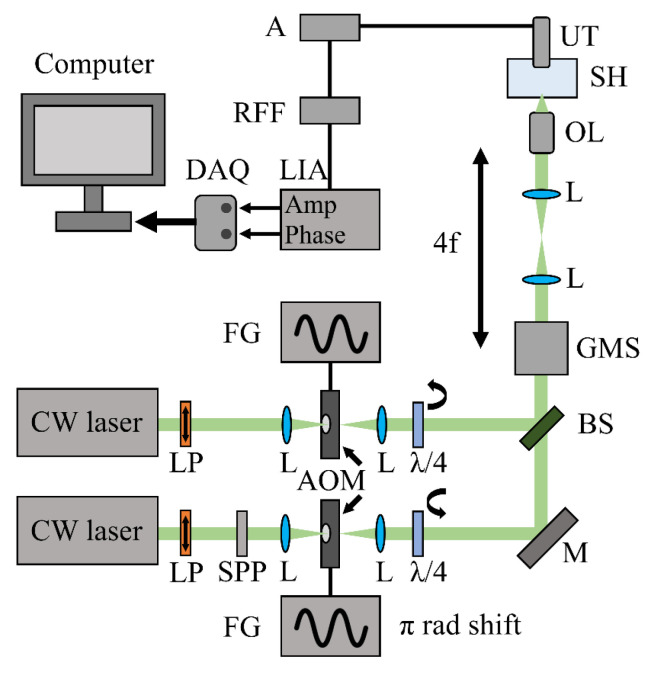
Proposed FD-PAM system integrating a phase-shifted excitation scheme.

The principles presented here can be directly implemented in a novel phase-shifted dual-beam FD-PAM platform, offering sub-diffraction-limited lateral resolution and potentially enabling z-sectioning capabilities. The proposed setup (Figure 7) could integrate two continuous-wave (CW) lasers emitting Gaussian TEM_00_ beams of the same optical wavelength. Linear polarizers (LPs) filter out the non-polarized components, producing beams with well-defined polarization. The optical radiation from the second laser passes through a spiral phase plate (SPP) to generate a donut-shaped beam, which is then focused by a positive lens (L) through an acousto-optic modulator (AOM), while, in parallel, the Gaussian beam from the first laser is focused onto an identical AOM. The AOM devices are driven by two function generators (FGs) providing sinusoidal waveforms that are phase-locked with a π-radian phase shift between them. The two beams are subsequently collimated and pass through separate quarter-wave plates (λ/4), producing left- and right-handed circular polarizations, respectively, thereby eliminating any interference effects during subsequent spatial overlap. The beams are then combined in the same optical axis using a highly reflective mirror (M) and a 50:50 beamsplitter (BS) before being guided on a galvo-mirror scanner (GMS). A 4f telescope configuration is employed to further guide the beams through a high-numerical-aperture objective lens (OL), which focuses the overlapping excitation radiation onto the investigated specimen positioned at the optically transparent bottom of a water-filled sample holder (SH). The generated single-frequency photoacoustic waves are then detected by an immersed transducer (UT), amplified by a radio-frequency (RF) amplifier (A), and band-passed through an RF filter (RFF), before their detection by a lock-in amplifier (LIA), which estimates the amplitude and phase of the signals. Finally, the extracted values are digitized and recorded by a data acquisition card (DAQ) in synchronization with the raster scanning of the beams by the galvo mirrors of the microscope.

Several additional practical challenges have to be addressed for this proposed experimental realization. Specifically, precise spatial overlap and stable phase locking between the two beams are very crucial for maintaining the excitation confinement, while imperfections in beam shaping, phase stability, or optical alignment could potentially degrade resolution enhancement. These considerations underline the importance of careful optical–acoustic design in future implementations. The experimental validation of the proposed concept could extend the application of photoacoustic microscopy systems to resolution-critical application areas of biomedical imaging, such as visualization of subcellular components (i.e., nuclei and lipid droplets) [21], label-free tracking of microscopic model organisms’ development [22], detailed mapping of microvasculature and oxygen dynamics [23], and detection of pathological changes, including tumor angiogenesis [24]. Further optimization of beam profiles (e.g., higher-order Gaussian–Laguerre beams) [25], modulation schemes, and detection strategies may enable additional improvements in terms of resolution and SNR performance. The weak residual ring artifacts arising from the resulting photoacoustic excitation profile could be further suppressed using image deconvolution techniques that account for the point spread function of the system [26]. Moreover, it has to be emphasized that the 10% side-lobe suppression level is used here as an indicative reference value, providing a practical compromise between effective reduction in the excitation region and suppression of peripheral ringing artifacts; alternative thresholds may be selected depending on application-specific requirements, as illustrated by the trade-off trends shown in Figure 4. These commonly used post-processing methods could further enhance image clarity and contrast without altering the introduced excitation scheme. Furthermore, the integration of the proposed excitation strategy with fast scanning mechanisms, multi-wavelength excitation [27,28], or advanced signal processing techniques such as machine learning algorithms [29,30,31] could additionally broaden its applicability. Overall, the present work provides a foundation for experimental realization of cost-efficient, resolution-enhanced frequency-domain photoacoustic microscopy systems. Further optimization and verification may translate the simulated lateral resolution gain to practical advancements towards high-definition, inexpensive biomedical imaging.

## Data Availability

The simulation codes presented in the study are openly available in the Open Science Framework repository at https://osf.io/ej9pa/overview?view_only=860ceaca9efa4ea4afb4c6947b4f17d9. Accessed on 11 January 2026.

## Supplementary Materials

The following supporting information can be downloaded at https://www.mdpi.com/article/10.3390/s26020498/s1: Video S1: π-rad phase-shifted intensity modulation at 10 MHz for a Gaussian and a donut-shaped beam. The sum of intensities is shown in the third graph.
